# Exogenous Methyl Jasmonate Mediated MiRNA-mRNA Network Improves Heat Tolerance of Perennial Ryegrass

**DOI:** 10.3390/ijms241311085

**Published:** 2023-07-04

**Authors:** Zongchao Liao, Hossein Ghanizadeh, Xin Zhang, Hechuan Yang, Ying Zhou, Linkai Huang, Xinquan Zhang, Yiwei Jiang, Gang Nie

**Affiliations:** 1 College of Grassland Science and Technology, Sichuan Agricultural University, Chengdu 611130, China; 2 School of Agriculture and Environment, Massey University, Palmerston North 4442, New Zealand; 3 Department of Agronomy, Purdue University, West Lafayette, IN 47907, USA; yjiang@purdue.edu

**Keywords:** high temperature, plant hormone, *Lolium perenne*, miRNA, target genes, regulatory mechanism

## Abstract

Heat stress can hinder the growth of perennial ryegrass (*Lolium perenne* L.). Methyl jasmonate (MeJA) applied exogenously can increase heat stress tolerance in plants; however, the regulatory mechanisms involved in heat tolerance mediated by MeJA are poorly understood in perennial ryegrass. Here, the microRNA (miRNA) expression profiles of perennial ryegrass were assessed to elucidate the regulatory pathways associated with heat tolerance induced by MeJA. Plants were subjected to four treatments, namely, control (CK), MeJA pre-treatment (T), heat stress treatment (H), and MeJA pre-treatment + heat stress (TH). According to the results, 102 miRNAs were up-regulated in all treatments, with 20, 27, and 33 miRNAs being up-regulated in the T, H, and TH treatment groups, respectively. The co-expression network analysis between the deferentially expressed miRNAs and their corresponding target genes showed that 20 miRNAs modulated 51 potential target genes. Notably, the miRNAs that targeted genes related to with regards to heat tolerance were driven by MeJA, and they were involved in four pathways: novel-m0258-5p mediated signal transduction, novel-m0350-5p mediated protein homeostasis, miR397-z, miR5658-z, and novel-m0008-5p involved in cell wall component, and miR1144-z and miR5185-z dominated chlorophyll degradation. Overall, the findings of this research paved the way for more research into the heat tolerance mechanism in perennial ryegrass and provided a theoretical foundation for developing cultivars with enhanced heat tolerance.

## 1. Introduction

Perennial ryegrass (*Lolium perenne* L.) is a forage grass widely cultivated in temperate regions and is the primary feed source for producing over 70% of the world’s cattle products [1,2]. Perennial ryegrass is also an ideal grass species for turfgrass use due to its quick establishment and high traffic tolerance [3,4]. Perennial ryegrass benefits ecosystems by boosting carbon uptake, preserving soil, and enhancing nutrient cycling [5]. However, this species is heat-sensitive, and temperatures above 27 °C often reduce its yield and quality [6]. Hence, heat stress has become one of the most challenging abiotic stresses that negatively impacts the growth and development of perennial ryegrass [7]. Breeding for heat-tolerant cultivars [6,8] or inducing heat tolerance by exogenous application of growth regulators have been proposed as effective strategies for enhancing perennial ryegrass heat stress tolerance [9,10].

Plant hormones are crucial in sensing, transmitting signals, and triggering proper responses to environmental stresses [11]. The phytohormone jasmonic acid (JA) and its related derivatives, such as methyl ester (MeJA) and isoleucine conjugate (JA-Ile), are well known signaling molecules that can regulate plant stress responses [10]. Several studies have demonstrated that JAs’ pre-treatment can prevent heat stress damage in plants through modifying osmotic regulation, anti-oxidative protection, and JA-mediated gene expression [9,10,12,13]. For instance, MeJA application during rice (*Oryza sativa* L.) anthesis reduced the buildup of reactive oxygen species, increased stigma vitality, and consequently promoted fertilization and seed-setting rates under heat stress [13]. Moreover, the exogenous application of MeJA during the booting stage mitigated the negative effects of heat stress by boosting the antioxidant and photosynthetic capacities of rice [14]. Recently, it was reported that exogenous application of MeJA improved heat tolerance in perennial ryegrass through modulating various biological pathways, such as JAs and chlorophyll biosynthesis and the heat shock factor (HSF)–heat shock protein (HSP) network [9]. However, post-transcriptional regulations underlying interactions between the heat response pathways and JAs are still unknown in perennial ryegrass.

MiRNAs (microRNAs) are non-coding small regulatory RNAs that modulate post-transcriptional regulations by promoting mRNA degradation [15,16,17]. MiRNAs have been proved to play a pivotal role in plants experiencing environmental stresses, including heat stress [16,18,19,20]. For instance, several mRNA–miRNA networks were found to be associated with mediating regulatory pathways underlying heat tolerance mechanisms in tomato (*Lycopersicon esculentum* Miller), cucumber (*Cucumis sativus* L.), and rice [19,21,22]. In perennial ryegrass, it was shown that overexpressing Os-miR408 improved heat tolerance in this species [6], indicating the crucial function of some miRNAs in improving heat tolerance.

Despite prior reports indicating that MeJA is essential in enhancing heat tolerance in perennial ryegrass, the underlying MeJA-mediated miRNA–mRNA regulatory network has remained largely unknown. Investigating miRNA expression in MeJA-treated perennial ryegrass can enlarge our comprehension of the molecular basis of heat tolerance mechanisms in this species. Hence, the objectives of this research were: to (1) identify differentially expressed miRNAs (abbreviated to DEMIRs) and their differentially expressed target genes (abbreviated to DEMTGs) under heat stress in perennial ryegrass pre-treated with MeJA; and (2) to identify transcriptional and post-transcriptional characteristics of key candidate genes related to heat resistance induced by MeJA in perennial ryegrass. The findings of this research can help uncover potential MeJA-mediated pathways involved in heat tolerance and provide a theoretical foundation for improving heat resilience in perennial ryegrass and related perennial grass species.

## 2. Results

### 2.1. RNA Sequencing and the Identification of Differentially Expressed miRNAs (DEMIRs)

There were three biological replicates for each of the four treatment groups, and a total of 12 small RNA libraries were constructed and sequenced using the Illumina Novaseq6000 platform. High correlations in miRNA expression profiles were observed among the biological replicates, showcasing the superior quality of the data (Figure S1). After filtering and processing the raw reads, a total of 11,570,005, 11,665,361, 15,974,703, and 16,350,105 clean reads were generated from CK, H, T, and TH treatment groups, respectively (Table S1). Clean reads, ranging in size from 15 to 35 nt, were obtained, and the length distribution of the clean reads in each library is presented in Figure S2. Blasting the clean reads against the miRBase database (Release 22) identified a total of 387 and 697 known and novel miRNAs, respectively (Tables S2 and S3).

**Figure 1:**
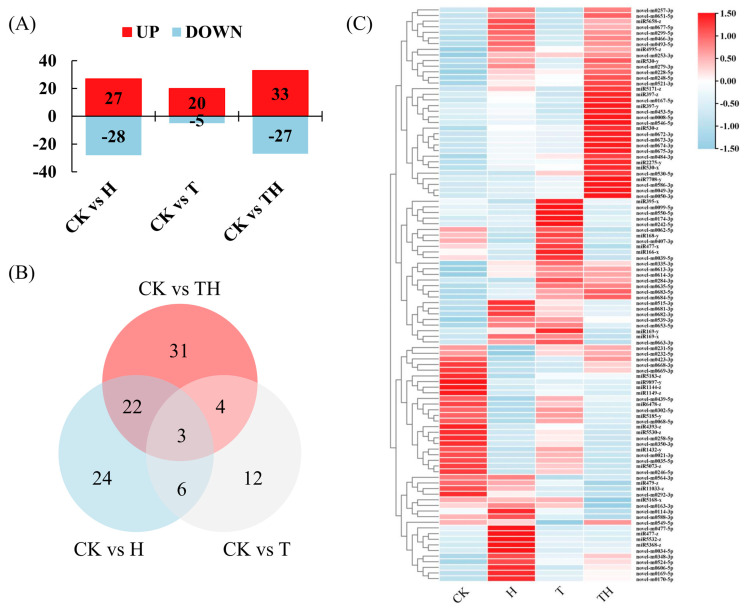
The differentially expressed miRNAs (DEMIRs) profile in response to the high temperature and exogenous application of methyl jasmonate (MeJA). (**A**) The column diagram represents the numbers of up- and down-regulated DEMIRs. (**B**) Venn diagrams illustrate the numbers of DEMIRs, and the overlaps of sets were obtained across three comparison pairs. (**C**) The heatmaps represent all DEMIR expression profiles under four treatments. All *p*-values were less than 0.05.

The expression of the known and novel differentially expressed miRNAs (DEMIRs) in the H-, T-, and TH-treated perennial ryegrass relative to the control (CK) was assessed using normalized read counts (TPM), and miRNAs with a fold change > 2 and a *p*-value ≤ 0.05 were considered differentially expressed. The results showed that a total of 102 DEMIRs were obtained, including 27, 20, and 33 up-regulated genes and 28, 5, and 27 down-regulated genes in the H, T, and TH treated plants, respectively (Figure 1A). The number of DEMIRs shared among the three treatments is demonstrated graphically using a Venn diagram in Figure 1B. The results showed that 25 DEMIRs (i.e., miR1144-z, miR1149-z, miR1432-y, miR166-x, miR168-y, miR4393-z, miR5073-z, miR5171-z, miR5185-y, miR530-y, miR5530-z, miR9897-y, novel-m0021-3p, novel-m0035-5p, novel-m0062-5p, novel-m0167-5p, novel-m0246-5p, novel-m0253-3p, novel-m0257-3p, novel-m0258-5p, novel-m0302-5p, novel-m0350-3p, novel-m0466-3p, novel-m0493-5p and novel-m0677-5p) were shared between the H and TH treatment groups. Seven DEMIRs (i.e., miR1144-z, miR530-x, miR9897-y, novel-m0253-3p, novel-m0484-3p, novel-m0521-3p and novel-m0635-5p------) were shared between the T and TH treatment groups, and nine DEMIRs (i.e., miR1144-z, miR169-x, miR395-x, miR4995-z, miR9897-y, novel-m0253-3p, novel-m0335-3p, novel-m0668-3p and novel-m0669-3p) were shared between the H and T treatment groups. It was, however, noted that the miR1144-z, miR9897-y and novel-m0253-3p candidates were shared among the H, T, and TH treatment groups (Figure 1B and Figure S3). The levels of DEMIR expression shown in all treatments are also illustrated in Figure 1C.

### 2.2. Functional Analysis of Differentially Expressed miRNAs (DEMIRs)

To gain insight into the biological functions of DEMIRs, the prediction of the target gene of miRNAs was carried out using the miRNA databases, and then functional and pathway enrichment analysis were carried out through the Gene Ontology (GO) and Kyoto Encyclopedia of Genes and Genomes (KEGG) databases. The top 20 GO terms and KEGG pathways with the smallest *q*-values were identified across all treatments. The GO terms were categorized into biological process, cellular component, and molecular function (Figure S4). The five GO terms with the smallest *q*-values are listed in Table 1. The GO terms of tyrosyl-DNA phosphodiesterase activity (GO:0070259) and phosphoric diester hydrolase activity (GO:0008081) were enriched for both the H and TH treatment groups. In addition, the GO terms of the metabolic hormone process (GO:0042445), regulation of biological quality (GO:0065008), and regulation of hormone levels (GO:0010817) were enriched for the TH treatment group. Most notably, 11 genes were enriched in the lignin metabolic process (GO:0009808), and 18 genes were enriched in the phenylpropanoid metabolic process (GO:0009698) for the TH treatment group.

A total of three, five, and twelve KEGG pathways were obtained for the H, T, and TH treatment groups, respectively (Table 2). The H treatment group had a relatively higher richness in plant–pathogen interaction (ko04626), RNA transport (ko03013), and DNA replication (ko03030) pathways. The pathways related to the tryptophan metabolism (ko00380), MAPK signaling pathways (ko04016), plant hormone signal transduction (ko04075), spliceosome (ko03040), and diterpenoid biosynthesis (ko00904) were highly enriched for the T treatment group. The KEGG pathway analysis, however, revealed a notably greater richness in the fatty acid elongation (ko00062), plant–pathogen interaction (ko04626), tryptophan metabolism (ko00380), fatty acid metabolism (ko01212), biosynthesis of unsaturated fatty acids (ko01040), stilbenoid, diarylheptanoid and gingerol biosynthesis (ko00945), peroxisome (ko04146), galactose metabolism (ko00052), pentose phosphate pathway (ko00030), pentose phosphate pathway (ko00030), fructose and mannose metabolism (ko00051), amino sugar and nucleotide sugar metabolism (ko00520), and ABC-transporter (ko02010) pathways for the TH treatment group. Greater enriched KEGG pathways for the TH treatment group may indicate that heat tolerance in MeJA-treated perennial ryegrass is regulated by a complex process.

### 2.3. Construction of the Co-Expression Network between DEMIRs and DEMTGs

To elucidate the regulation network of miRNAs–mRNA (miRNAs–targets/target genes), the miRNAs-regulated target genes that were differentially expressed (DEMTGs) (Figure S5A) and overlapped with the predicted target gene from the previously published perennial ryegrass mRNA sequences database were identified. The results showed that 258, 153, and 240 DEMTGs were up-regulated, and 134, 227, and 149 DEMTGs were down-regulated in the H, T, and TH treatment groups, respectively (Figure S5B). All three treatments shared 221 DEMTGs (Figure S5C). Nevertheless, eight, four, and three genes were uniquely expressed in the T, H, and TH treatment groups, respectively (Table S4).

**Figure 2:**
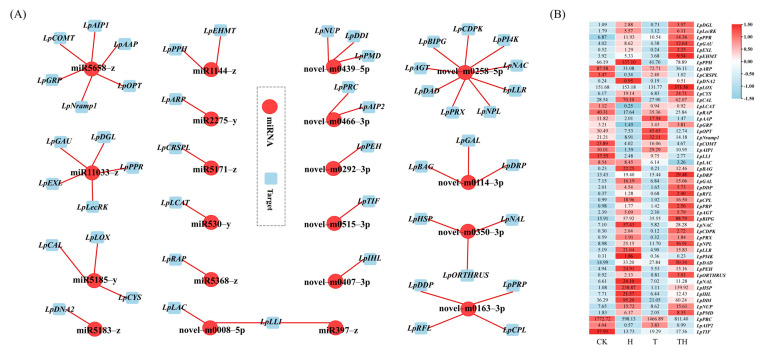
The miRNA–targets correlation network. (**A**) The miRNA–target genes correlation network. The miRNA ID is in red circles, and the target ID is in blue squares. The red straight line indicates miRNA negatively regulated targets. (**B**) The heatmap of target genes expression level. The numbers representing the expression level (TPM) recorded in the RNA sequencing.

The expression correlation between miRNA and target genes was measured using Pearson’s correlation coefficient (PCC) to elaborate the miRNA–target network. The miRNA–target network was then generated and visualized using the Cytoscape software (Figure 2A,B). A total of 20 DEMIRs, regulating 51 target genes, were constructed in the network, of which 10 were novel DEMIRs, regulating 29 target genes. Among the novel DEMIRs, the novel-m0258-5p was found to regulate the maximum number of target genes, followed by the novel-m0163-3p. The results also showed that the novel-m0114-3p, novel-m0350-3p, and novel-m0439-5p each regulated three different target genes. Except for the novel-m0466-3p and novel-m0008-5p, which regulated two target genes, the other novel DEMIRs regulated a single gene. It was noted that the novel-m0008-5p down-regulated two cell wall laccase-associated genes, *LpLLI* and *LpLAC*, in the TH treatment group (gene ID was simplified in Table S6). In addition, it was noted that the *LpLLI* was jointly regulated by both the novel-m0008-5p and miR397-z.

Among the known DEMIRs, the miR5658-z regulated the greatest number of target genes (n = 6), including *LpCOMT* (Figure 2A,B), followed by miR11033-z, regulating five unique genes. miR5185-y and miR1144-z each regulated three and two target genes, respectively. The other known DEMIRs were found to regulate a single target gene.

### 2.4. Analysis of Key Pathways Responding to Heat Stress and Exogenous Methyl Jasmonate

**Figure 3:**
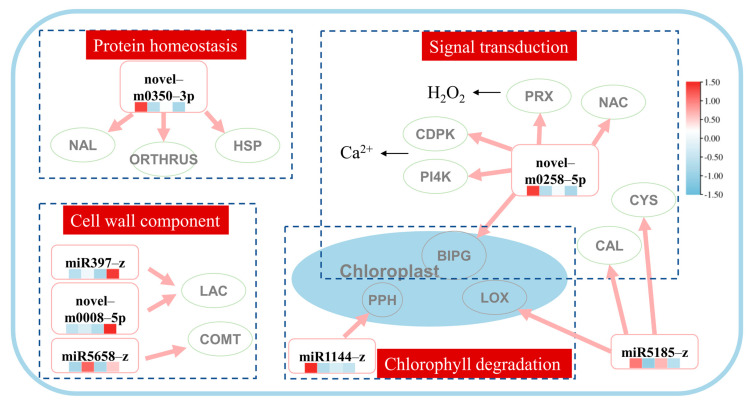
Prediction miRNA–mRNA network of heat tolerance in perennial ryegrass with exogenous methyl jasmonate application. The heatmap under miRNA ID represented the miRNA expression level in CK, T, H, and TH groups and TPM was row scale normalized. In the protein homeostasis pathway, novel-m0350-5p regulated *the NAC domain-containing protein 71-like* (*LpNAL*), *heat shock protein 90* (*LpHSP*), and *E3 ubiquitin-protein ligase ORTHRUS 2-like* (*LpORTHRUS*) genes. In the cell wall component pathway, miR397-z and novel-m0008-5p co-mediated two *laccase* genes (*LpLAC*), and miR5658-z mediated the *caffeic acid O-methyltransferase* gene (*LpCOMT*). In the signal transduction pathway, the expressions of the *peroxidase 4-like* (*LpPRX*), *NAC transcription factor* (*LpNAC*), *GTP-binding protein BRASSINAZOLE INSENSITIVE PALE GREEN 2, phosphatidylinositol 4-kinase gamma* (*LpPI4K*), *calcium-dependent protein kinase* (*LpCDPK*), and *chloroplast-related* (*LpBIPG*) genes were mediated by the novel-m0258-5p and miR5185-z mediated the *calmodulin-binding receptor-like cytoplasmic kinase* (*LpCAL*) and *cysteine-rich receptor-like protein kinase* (*LpCYS*) genes. Lastly, in the chloroplast degradation pathway, the expressions of the *pheophorbide A oxygenase* (*LpPPH*), *lipoxygenase 2.3* (*LpLOX*), and *LpBIPG* genes were regulated by miR1144-z, miR5185-z, and novel-m0258-5p, respectively.

To further identify key pathways in response to heat stress and exogenous application of MeJA, we selected key pathways associated with heat stress and exogenous MeJA, which are based on the expression level of the genes identified in the previous section. A hypothetical model, including four miRNA–target pathways, signal transduction, protein homeostasis, cell wall component, and chlorophyll degradation, was established (Figure 3). In the signal transduction pathway, the expressions of the *peroxidase 4-like* (*LpPRX*), *NAC transcription factor* (*LpNAC*), *GTP-binding protein BRASSINAZOLE INSENSITIVE PALE GREEN 2, phosphatidylinositol 4-kinase gamma* (*LpPI4K*), *calcium-dependent protein kinase* (*LpCDPK*), and *chloroplast-related* (*LpBIPG*) genes were mediated by the novel-m0258-5p. It was also noted that miR5185-z mediated the *calmodulin-binding receptor-like cytoplasmic kinase* (*LpCAL*) and *cysteine-rich receptor-like protein kinase* (*LpCYS*) genes in the signal transduction pathway. In the protein homeostasis pathway, *the NAC domain-containing protein 71-like* (*LpNAL*), *heat shock protein 90* (*LpHSP*), and *E3 ubiquitin-protein ligase ORTHRUS 2-like* (*LpORTHRUS*) genes were mediated by the novel-m350-3p. In the pathways associated with the cell wall balance, the miR397-z and novel-m0008-5p co-mediated two *laccase* genes (*LpLAC*), and miR5658-z mediated the *caffeic acid O-methyltransferase* gene (*LpCOMT*). In the chloroplast degradation pathway, the expressions of the *pheophorbide A oxygenase* (*LpPPH*), *lipoxygenase 2.3* (*LpLOX*), and *LpBIPG* genes were regulated by the miR1144-z, miR5185-z, and novel-m0258-5p, respectively.

### 2.5. Expression Verification of Differential miRNAs and Target Genes by qRT-PCR

**Figure 4:**
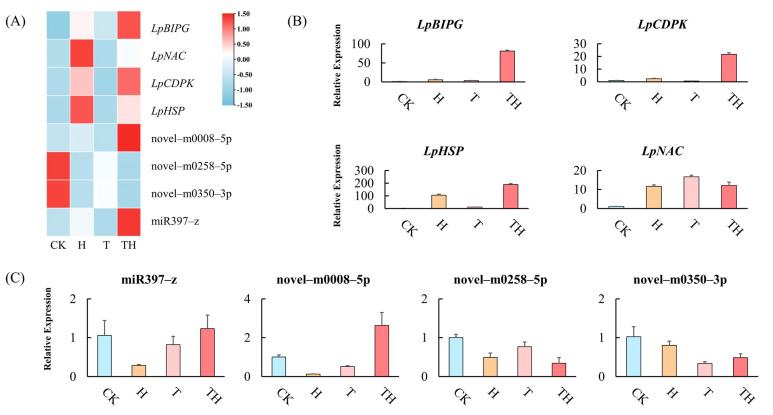
The expression analysis of miRNAs and target genes. (**A**) The expression of miRNAs and target genes in the sequencing data. (**B**) The expression of target genes measured by the qRT-PCR analysis. (**C**) The expression of miRNAs measured by qRT-PCR analysis. Error bars indicate ± standard deviation of three biological replicates.

In order to validate the expression model from mRNA and miRNA sequencing, four miRNAs (miR397-z, novel-m0008-5p, novel-m0258-5p, and novel-m0350-3p) and four target genes (*LpBIPG*, *LpCDPK*, *LpHSP*, and *LpNAC*) were selected randomly. The expressions of miRNAs and target genes were quantified by qRT-PCR (Figure 4A). The results showed that the expression of the selected miRNAs and target genes was consistent between the sequencing data and the qRT-PCR analysis. For example, the high expression of *LpBIPG* and *LpCDPK* were detected in the TH treatment group compared to the other treatment group (Figure 4B). Furthermore, novel-m0008-5p was up-regulated in the TH treatment group (Figure 4C).

## 3. Discussion

Although there is substantial evidence linking miRNAs to the response of plants to stresses, our grasp of this relationship is still far from complete. Additionally, efforts to increase plants’ stress tolerance are restricted by the paucity of published research on the expression of miRNA-controlling genes related to stress tolerance induced by the exogenous treatment of phytohormones. Previously, it was shown that miRNAs can regulate plant response to heat stress [21], and MeJA can validly mediate the expression of miRNAs in plants experiencing heat stress [23,24,25,26]. In this study, 20 responsive miRNAs were identified in MeJA pre-treated perennial ryegrass under heat stress, suggesting a role of these miRNAs in the improvement of heat tolerance mediated by MeJA. It has been demonstrated that exogenous application of MeJA improved heat tolerance in perennial ryegrass through alterations in osmotic regulation, anti-oxidative protection, and JA-mediated gene expression [10]. However, the regulatory pathways associated with heat stress tolerance induced by MeJA treatment in this species were still unknown. As several studies have highlighted that miRNAs are essential regulators of genes associated with stress tolerance induced by MeJA treatment, we assessed putative MeJA-mediated miRNA pathways, mediating heat stress tolerance in perennial ryegrass. For this, we assessed the DEMIRs in the MeJA-treated and untreated plants under heat stress and constructed the co-expression network of the DEMIRs and their corresponding mRNAs to elucidate the mechanism underlying heat stress tolerance induced by the MeJA pre-treatment. Using this approach, we revealed that heat stress tolerance induced by MeJA in perennial ryegrass is associated with four key pathways: signal transduction, protein homeostasis, the cell wall component, and chlorophyll degradation pathways (Figure 3).

The signal transduction pathway initiates a cascade of signals in response to stress, leading to plant response to stress [27]. Heat stress signaling pathways in plants involve secondary messengers, such as calcium ions (Ca^2+^), reactive oxygen species (ROS), and inositol phosphatases [28,29]. Heat stress generally changes the physical state of membranes by influencing membrane proteins, permeability, and structure [27]. Membrane-associated molecules detect and activate lipid-signaling molecules in response to abnormalities in the membrane induced by high temperatures. This induced the activation of calcium-dependent protein kinases (CDPKs) and phosphatidylinositol 4-kinase (PI4K), allowing inward Ca^2+^ influx into the cytoplasm [30,31]. The results of this research showed that the novel-m0258-5p candidate was down-regulated under heat stress (Figure 4A), and its target genes, the *calcium-dependent protein kinase gene* (*LpCDPK*) and *phosphatidylinositol 4-kinase gamma 4 gene* (*LpPI4K*), were up-regulated in ROS pre-treated plants under heat stress (TH treatment) (Figure 2). These results indicated that MeJA promoted miRNA-mediated signaling molecules to enhance heat tolerance in perennial ryegrass.

Hydrogen peroxide (H_2_O_2_), as a reactive oxygen species (ROS), was formed in a redox reaction catalyzed by oxidase, but hydrogen was catalyzed by peroxidase [32]. The up-regulation of the peroxidase gene is a defense mechanism to prevent cell damage caused by H_2_O_2_ buildup in stressed plants [33]. In this study, the *peroxidase 4-like gene* (*LpPRX*), mediated by the novel-m0258-5p, was up-regulated in the H and TH treatment groups, though the level of expression of this gene in the TH treatment group was lower than that in the H treatment group, indicating that MeJA protected the TH-treated plants against the excessive accumulation of H_2_O_2_ under heat stress (Figure 2).

Transcription factors and protein kinases are crucial components of the signal transduction networks that connect the stress signal perception to the expression of genes involved in stress response [34,35]. In this study, it was noted that miR5185-z regulated the *cysteine-rich receptor-like protein kinase 6* (*LpCYS*) gene, which is a large subfamily of receptor-like protein kinases [36]. It was also noted that the expression level of the *LpCYS* gene was higher in the TH treatment group compared to the heat stress treatment (H). Moreover, it was found that the *NAC transcription factor 4 gene* (*LpNAC*) regulated by the novel-m0258-5p can have a role in heat-response signaling pathways, since this gene was up-regulated in the H treatment group, while it was down-regulated in the TH treatment group (Figure 2). The NAC gene family members encode transcription factors that are vital in controlling transcriptional reprogramming related to plant stress responses [37]. For instance, a NAC-type transcription factor, CaNAC2c, suppressed H_2_O_2_ accumulation in response to heat stress in *Capsicum annuum* by inducing the heat-shock protein A5 (CaHSFA5) transcription [35]. In addition, it has been demonstrated that the NAC transcription factor mediates the expression of HsfA1s and Hsfs transcription factors, which induce the expression of heat-stimulated proteins (HSPs) [38]. HSPs encode molecular chaperones that denature excess proteins accumulated during heat stress to maintain cell protein homoeostasis [39,40]. In this study, the *NAC domain-containing protein 71-like* (*LpNAL*) and *heat shock protein 90* (*LpHSP*) genes were up-regulated under heat stress, but down-regulated after MeJA pre-treatment (Figure 2), indicating a potential role of this gene in heat tolerance. Additionally, an E3 ubiquitin-protein ligase, *LpORTHRUS,* was up-regulated under heat stress, though the level of expression of this gene in the TH treatment group was greater than in the H treatment group (Figure 2). E3 ubiquitin-protein ligase is essential for the ubiquitin–proteasome pathway, one of the most important pathways for protein degradation [41]. In response to heat stress, plasma membrane-localized E3 ligase TT3.1 safeguards thylakoids by ubiquitinating chloroplast precursor protein TT3.2 [41]. This implies that the primary function of the novelm0350-3p is to maintain protein homeostasis under heat stress by mediating the *LpNAL*, *LpHSP,* and *LpORTHRUS* genes, which are involved in excess protein degradation (Figure 3).

The results of this research revealed that MeJA pre-treatment increased the expression of miRNAs, modulating genes associated with cell wall lignin under heat stress (Figure 2 and Figure 3). Overexpression of *OsmiR408* has been shown to increase thermotolerance in perennial ryegrass by inhibiting the expression of the *laccase* (*LAC3*) gene [6]. In this research, it was noted that the expression of *laccase* genes (*LpLAC* and *LpLLI*) was controlled by the miR397 and novel-m0008-5p in plants pre-treated with MeJA under heat stress (Figure 2). However, the expression of *LpLAC* was down-regulated in TH treatment, while *LpLLI* was up-regulated compared to the H treatment, implying that the differential expression patterns of different laccase genes affect the composition of lignin, thereby affecting the function of cell walls [42]. It is likely that MeJA promotes the expression of miR397 and novel-m0008-5p to enhance heat tolerance by preventing the breakdown of lignin by *laccase* (Figure 2). It was also noted that miR5658-z up-regulated *LpCOMT*, which encodes the caffeic acid O-methyltransferase enzyme (COMT), an essential enzyme for lignin synthesis [43,44,45,46]. Previous studies have shown that knocking down the *COMT* gene resulted in a more severe reduction in photosynthesis under heat stress, suggesting that COMT has a crucial role in heat tolerance [47].

Degradation of chlorophyll is a common negative consequence of heat stress [48]. Exogenous MeJA could induce the expression of genes involved in chlorophyll (Chl) biosynthesis and degradation [9]. In this study, plants pre-treated with MeJA showed increased expression of miR1144-z, which modulates genes related to chlorophyll degradation under heat stress (Figure 2). Based on the results, miR1144-z mediated the *LpPPH* gene, which was significantly down-regulated in the TH treatment group. It has been shown that the overexpression of the *PPH* gene accelerates chlorophyll degradation [49]. Hence, the down-regulation of the *PPH* gene in the TH treatment suggests that the addition of MeJA greatly alleviated the heat damage to chlorophyll. In addition, the miR5185-z and novel-m0258-5p candidates were found to mediate the *LpLOX* (*chloroplastic lipoxygenase*) and *LpBIPG* (*chloroplastic BRASSINAZOLE INSENSITIVE PALE GREEN 2*) genes (Figure 2 and Figure 3). *Lipoxygenase* (*LOX*) has an essential role in conferring biotic and abiotic stress resistance in plants by producing protective components, such as jasmonates, divinyl ethers, and leaf aldehydes [50,51]. In this study, the chloroplast *LpLOX* gene was highly up-regulated by the miR5185-z in the TH treatment group, indicating that the application of MeJA enhanced the *LpLOX* gene expression level under heat stress in perennial ryegrass. The *LpBIPG* gene was also up-regulated by the novel-m0258-5p under heat stress, with the plants in the TH treatment group exhibiting higher expression levels of this gene (Figure 2). The *BIPG* gene encodes a GTP-binding protein involved in chloroplast differentiation and the accumulation of chloroplast rRNA through its involvement in brassinosteroid-mediated post-transcriptional and translational control [52,53]. Our results supported that the application of MeJA improved heat tolerance in perennial ryegrass, possibly through regulation of *LpBIPG* for maintaining chloroplast differentiation and function.

## 4. Materials and Methods

### 4.1. Plant Materials and Treatments

Five hundred seeds (1 g) of perennial ryegrass (*Lolium perenne* L.), cultivar ‘Esquire’ (DLF Seeds A/S Co., Beijing, China), were sown in plastic containers filled with quartz sand that were 20 cm long, 15 cm wide, and 10 cm high. The containers were placed in a greenhouse at 20 °C/15 °C (12 h of daylight and 12 h of night), 70% relative humidity, and 200 μmol m^−2^·s^−1^ light intensity, and daily irrigation was performed with distilled water. Seven days after seedling emergence, the containers were watered every other day with 1× Hoagland nutrient solution (Hopebio Co., Ltd., Beijing, China). After 30 days, when the plants were at the five-leaf stage, they were split into two groups. The first group was irrigated with a 30 mL of 100 μM of MeJA solution (Beijing Solarbio Science & Technology Co., Ltd., Beijing, China) dissolved in 1× Hoagland nutrient solution, while the other group was only irrigated with 1× Hoagland nutrient solution. The plants in each group were irrigated thrice with their corresponding solution every other day for 7 days. Subsequently, half of each group’s plants were exposed to heat stress (38 °C) for 12 h, while the other half were maintained at 20 °C (optimal condition). Therefore, the experiment included four treatment groups, including the (1) control group (CK), in which plants were not subjected to MeJA pre-treatment and heat stress; (2) MeJA-only pre-treatment (T), in which plants were only treated with the MeJA solution; (3) heat stress (H), in which plants were not pre-treated with the MeJA solution, but subjected to heat stress at 38 °C; and (4) MeJA pre-treatment and heat stress (TH), in which plants were first pre-treated with the MeJA solution and subsequently subjected to heat stress at 38 °C. Leaf samples from all four groups were collected 12 h after treatments. The collected leaf samples were immediately frozen using liquid nitrogen and kept in a −80 °C refrigerator. There were three biological replicates for each treatment.

### 4.2. RNA Extraction, Sequencing and Library Construction

Total RNA was extracted using a Trizol reagent kit (Invitrogen, Carlsbad, CA, USA), following the manufacturer’s instructions. To construct the miRNA-seq libraries, the small RNAs between 18 and 30 nt in length were enriched from total extracted RNA using polyacrylamide gel electrophoresis (PAGE). Subsequently, 3′ and 5′ adapters were ligated to the small RNAs. The ligated products were then subjected to reverse transcription polymerase chain reaction (RT-PCR). A cDNA library was created using PCR products with a size range of 140–160 bp and was then sequenced using an Illumina Novaseq6000 by Gene Denovo Biotechnology Co. (Guangzhou, China).

Raw sequencing data were filtered to obtain clean tags using fataq. The clean tags were produced by trimming the adaptors and removing low-quality reads. The read with a *q*-value < 20 or including unknown nucleotides (N) bases, or with a length less than 18 nt, was considered low quality and removed from further analysis. The clean tags were blasted against the Rfam database (Release 11.0, https://rfam.org/ (accessed on 2 July 2023)) and GenBank database (Release 209.0, https://www.ncbi.nlm.nih.gov/genbank/ (accessed on 2 July 2023)) to recognize and eliminate other non-coding RNAs. After removing the non-coding RNAs, the perennial ryegrass reference genome was used to map the clean tags using the bowtie. (version 1.1.2, https://bowtie-bio.sourceforge.net/manual.shtml (accessed on 2 July 2023)) [54]. The small RNA sequence data from this manuscript have been uploaded to the National Center for Biotechnology Information (NCBI) database (https://www.ncbi.nlm.nih.gov/ (accessed on 2 July 2023)), as shown by the bio-project accession number PRJNA768969.

### 4.3. Identification of miRNAs and Differential Expression Analysis of miRNAs

All clean tags were blasted against the miRBase database (Release 22.0, https://mirbase.org/ (accessed on 2 July 2023)) to detect known miRNAs. The unannotated tags were mapped to the ryegrass reference genome [54] to identify novel miRNA candidates. The novel miRNAs were determined, conforming to their genome locations and hairpin structures predicted by mirdeep2. Transcript per million (TPM) was used to calculate miRNA expression levels using the equation below:

TPM = Actual miRNA counts/Total clean tag counts × 10^6^

The number of genomic tags in each sample was utilized for determining the differentially expressed miRNAs (DEMIRs) using data from the TPM normalization. The DEMIRs among treatments were analyzed using edgeR. The miRNAs with a fold change > 2 and a *p*-value ≤ 0.05 were regarded differentially expressed between treatments. Heatmaps and Venn diagrams were created with TBtools to illustrate DEMIRs among treatments [55].

### 4.4. Target Gene Prediction, Gene Ontology (GO) and Kyoto Encyclopedia of Genes and Genomes (KEGG) Analysis

Target genes of miRNAs in response to heat stress and exogenous methyl jasmonate application were predicted using patmatch (Version 1.2, https://www.arabidopsis.org/cgibin/patmatch/nph-patmatch.pl (accessed on 2 July 2023)). The biological function and major biochemical and metabolic pathways of the candidate genes were identified using the gene ontology (GO) (http://geneontology.org/ (accessed on 2 July 2023)) and Kyoto Encyclopedia of Genes and Genomes (KEGG) (https://www.genome.jp/kegg/ (accessed on 2 July 2023)) databases. Using this approach, the candidate genes were classified into three categories: biological process, cellular component, and molecular function, and the functions of miRNAs were predicted based on their proximity to the genes in these three categories. Subsequently, we compared the target candidate genes to the perennial ryegrass reference genome [23] using a hypergeometric test to identify highly enriched GO terms in each group. The GO terms with a corrected *p*-value ≤ 0.05 were considered significantly enriched. Similarly, the target genes were assigned to KEGG terms, and a hypergeometric test was used to determine the significantly enriched KEGG metabolic pathways (corrected *p*-value ≤ 0.05) for the candidate target genes.

### 4.5. Co-Expression Network of miRNA-mRNA Construction

To better understand the miRNA–mRNA network, we compared the target gene identified in this study to the mRNA sequences identified previously by Nie et al. (NCBI Bioproject accession number = PRJNA766242) [9], with the overlaps serving as anticipated differential target genes of miRNAs (DEMTGs). Pearson’s correlation coefficient (PCC) was used to assess the expression correlation between miRNA and the target gene network, and pairs with *p*-value ≤ 0.05 and PCC < 0.7 were chosen as negatively co-expressed miRNA-target network pairs. The visualization of the miRNA-target networks was established using the Cytoscape software (v3.6.0, http://www.cytoscape.org/ (accessed on 2 July 2023)).

### 4.6. Verification of miRNA and Targets by qRT-PCR Analysis

The expression of four key candidate miRNAs and targets from the sequencing results were validated by quantitative real-time PCR (qRT-PCR) using the primers listed in Table S6. MiRNA 1st Strand cDNA Synthesis Kit (by stem-loop method) (Vazyme Biotech Co., Ltd., Nanjing, China.) and 5X All-In-One RT MasterMix with AccuRT (Applied Biological Materials Inc., Richmond, BC, Canada) were used for the RT-PCR analysis of the key miRNA and target genes, respectively, following the manufacturer’s instruction. MiRNA Universal SYBR qPCR Master Mix (Vazyme Biotech Co., Ltd., Nanjing, China.) and BlasTaq™ 2X qPCR MasterMix (Applied Biological Materials Inc., Richmond, BC, Canada) were used for the qRT-PCR analysis of the key miRNA and target genes, respectively. The internal references of U6 and eIF4A were used for normalization, respectively. The reaction system and qPCR cycling program were listed in Tables S7 and S8. The qRT-PCR analysis was performed using the Bio-Rad CFX96 Touch quantitative real-time PCR system (Bio-Rad, Hercules, CA, USA). The 2^−ΔΔCT^ approach was used to determine the relative expression levels of the key miRNAs and target genes [56]. The IBM SPSS Statistics for Windows, version 20.0 (IBMCorp., Armonk, NY, USA) was used to calculate the mean value and the standard deviation from three biological replicates.

## 5. Conclusions

This research identified miRNAs and their corresponding target genes associated with enhanced heat tolerance in perennial ryegrass when pre-treated with MeJA. The identified miRNAs (i.e., miR397-z, miR1144-z, miR5658-z, novel-m0008-5p, novel-m0258-5p, and novel-m0350-3p) regulated the expression of their corresponding target genes through four pathways (i.e., heat stress signal transduction, protein homeostasis, cell wall-associated genes, and chlorophyll degradation pathways). The knowledge of the miRNAs and their corresponding target genes aids in the understanding of the molecular basis of MeJA-mediated heat tolerance. This study revealed the miRNA regulatory network mediated by MeJA under heat stress and single miRNAs that can simultaneously regulate multiple target genes. Further work is necessary to validate this regulatory network and to explore the interaction between these target genes in perennial ryegrass.

## Figures and Tables

**Table 1:** The significantly enriched GO terms of genes targeted by miRNAs in response to high temperature and methyl jasmonate application.

	GO Terms	Description	Number of Genes	*q*-Value	Ontology
CK-H	GO:0070259	tyrosyl-DNA phosphodiesterase activity	7	1.71 × 10^−11^	Molecular Function
	GO:0008081	phosphoric diester hydrolase activity	7	7.48 × 10^−5^	Molecular Function
	GO:0005768	endosome	9	3.12 × 10^−4^	Cellular Component
	GO:0016020	membrane	105	1.62 × 10^−3^	Cellular Component
	GO:0042623	ATPase activity, coupled	19	9.77 × 10^−3^	Molecular Function
CK-T	GO:0042445	hormone metabolic process	7	1.87 × 10^−6^	Biological Process
	GO:0065008	regulation of biological quality	11	1.85 × 10^−5^	Biological Process
	GO:0010817	regulation of hormone levels	7	2.66 × 10^−5^	Biological Process
	GO:0009791	post-embryonic development	12	2.66 × 10^−5^	Biological Process
	GO:0018488	aryl-aldehyde oxidase activity	3	4.82 × 10^−5^	Molecular Function
CK-TH	GO:0070259	tyrosyl-DNA phosphodiesterase activity	7	1.66 × 10^−10^	Molecular Function
	GO:0009808	lignin metabolic process	11	5.99 × 10^−10^	Biological Process
	GO:0009698	phenylpropanoid metabolic process	18	4.75 × 10^−6^	Biological Process
	GO:0005576	extracellular region	26	1.57 × 10^−4^	Cellular Component
	GO:0008081	phosphoric diester hydrolase activity	7	6.36 × 10^−4^	Molecular Function

The pathways with a *q*-value (corrected *p*-value) < 0.05 were considered significantly enriched.

**Table 2:** The significantly enriched KEGG pathways of genes targeted by miRNAs responding to high temperature and methyl jasmonate application.

	Pathway ID	Pathway	Number of Genes
CK-H	ko04626	Plant–pathogen interaction	11
	ko03013	RNA transport	9
	ko03030	DNA replication	4
CK-T	ko00380	Tryptophan metabolism	3
	ko04016	MAPK signaling pathway-plant	2
	ko04075	Plant hormone signal transduction	2
	ko03040	Spliceosome	2
	ko00904	Diterpenoid biosynthesis	1
CK-TH	ko00062	Fatty acid elongation	4
	ko04626	Plant–pathogen interaction	11
	ko00380	Tryptophan metabolism	5
	ko01212	Fatty acid metabolism	7
	ko01040	Biosynthesis of unsaturated fatty acids	4
	ko00945	Stilbenoid, diarylheptanoid, and gingerol biosynthesis	3
	ko04146	Peroxisome	7
	ko00052	Galactose metabolism	5
	ko00030	Pentose phosphate pathway	4
	ko00051	Fructose and mannose metabolism	4
	ko00520	Amino sugar and nucleotide sugar metabolism	7
	ko02010	ABC transporters	5

The pathways with a *q*-value (corrected *p*-value) < 0.05 were considered significantly enriched.

## Data Availability

Sequencing data for perennial ryegrass are available on NCBI (PRJNA766242 and PRJNA768969).

## Supplementary Materials

The following supporting information can be downloaded at: https://www.mdpi.com/article/10.3390/ijms241311085/s1.
