# Supplementary material for: Exogenous Methyl Jasmonate Mediated MiRNA-mRNA Network Improves Heat Tolerance of Perennial Ryegrass
Source: Int J Mol Sci. 2023 Jul 4;24(13):11085. doi: 10.3390/ijms241311085 (PMC10341802; doi:10.3390/ijms241311085)
Supplement: Supplementary file 1 [file ijms-24-11085-s001.zip › Figure S3.pdf]

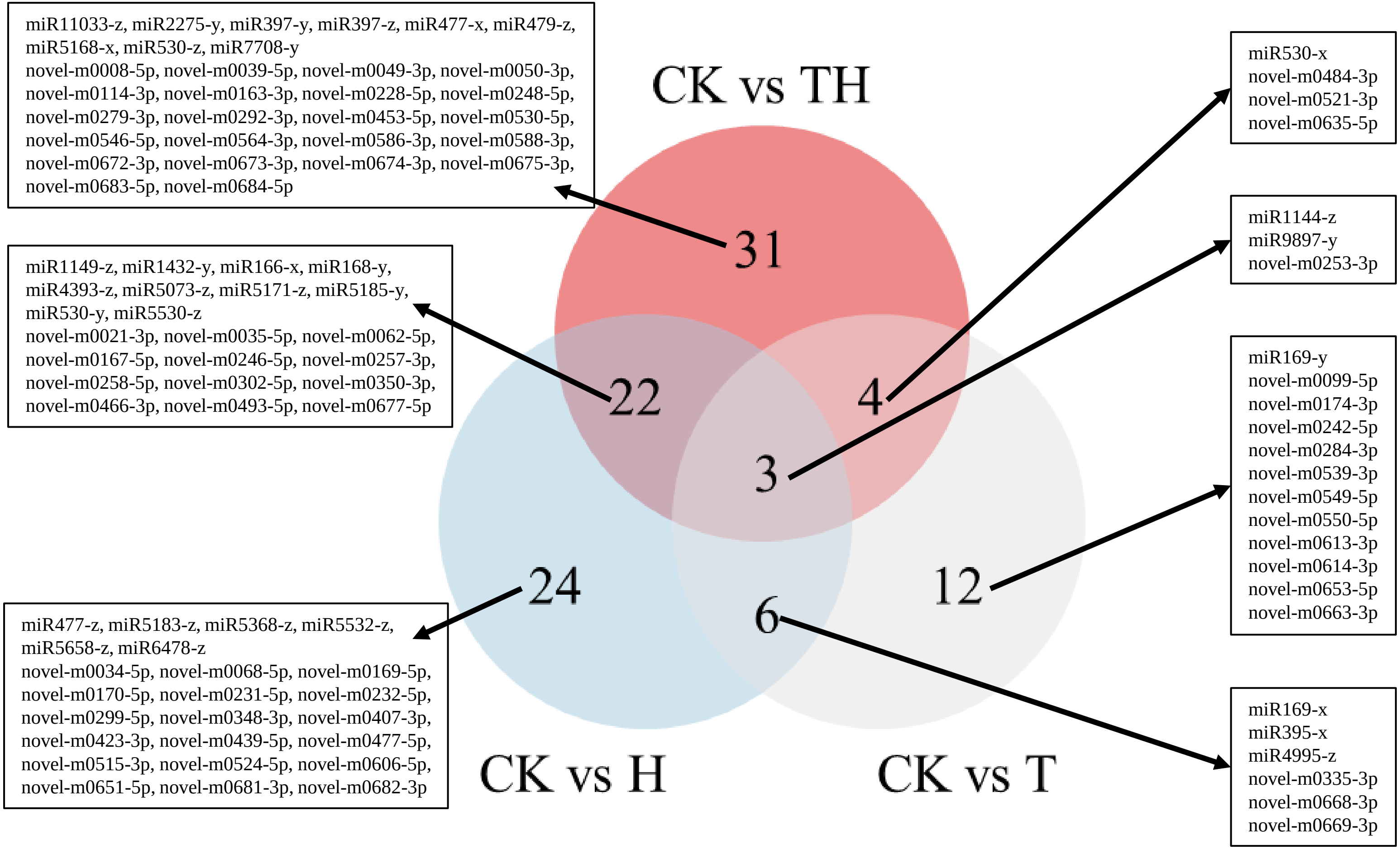
miR11033-z, miR2275-y, miR397-y, miR397-z, miR477-x, miR479-z, miR5168-x, miR530-z, miR7708-y
novel-m0008-5p, novel-m0039-5p, novel-m0049-3p, novel-m0050-3p, novel-m0114-3p, novel-m0163-3p, novel-m0228-5p, novel-m0248-5p, novel-m0279-3p, novel-m0292-3p, novel-m0453-5p, novel-m0530-5p, novel-m0546-5p, novel-m0564-3p, novel-m0586-3p, novel-m0588-3p, novel-m0672-3p, novel-m0673-3p, novel-m0674-3p, novel-m0675-3p, novel-m0683-5p, novel-m0684-5p
CK vs TH
miR530-x
novel-m0484-3p
novel-m0521-3p
novel-m0635-5p
31
miR1149-z, miR1432-y, miR166-x, miR168-y, miR4393-z, miR5073-z, miR5171-z, miR5185-y, miR530-y, miR5530-z
novel-m0021-3p, novel-m0035-5p, novel-m0062-5p, novel-m0167-5p, novel-m0246-5p, novel-m0257-3p, novel-m0258-5p, novel-m0302-5p, novel-m0350-3p, novel-m0466-3p, novel-m0493-5p, novel-m0677-5p
miR1144-z
miR9897-y
novel-m0253-3p
22
4
3
miR169-y
novel-m0099-5p
novel-m0174-3p
novel-m0242-5p
novel-m0284-3p
novel-m0539-3p
novel-m0549-5p
novel-m0550-5p
novel-m0613-3p
novel-m0614-3p
novel-m0653-5p
novel-m0663-3p
24
12
6
miR477-z, miR5183-z, miR5368-z, miR5532-z, miR5658-z, miR6478-z
novel-m0034-5p, novel-m0068-5p, novel-m0169-5p, novel-m0170-5p, novel-m0231-5p, novel-m0232-5p, novel-m0299-5p, novel-m0348-3p, novel-m0407-3p, novel-m0423-3p, novel-m0439-5p, novel-m0477-5p, novel-m0515-3p, novel-m0524-5p, novel-m0606-5p, novel-m0651-5p, novel-m0681-3p, novel-m0682-3p
miR169-x
miR395-x
miR4995-z
novel-m0335-3p
novel-m0668-3p
novel-m0669-3p
CK vs H
CK vs T
