# Supplementary figures and images for: Exogenous Methyl Jasmonate Mediated MiRNA-mRNA Network Improves Heat Tolerance of Perennial Ryegrass
Source: Int J Mol Sci. 2023 Jul 4;24(13):11085. doi: 10.3390/ijms241311085 (PMC10341802; doi:10.3390/ijms241311085)

Correlation heatmap

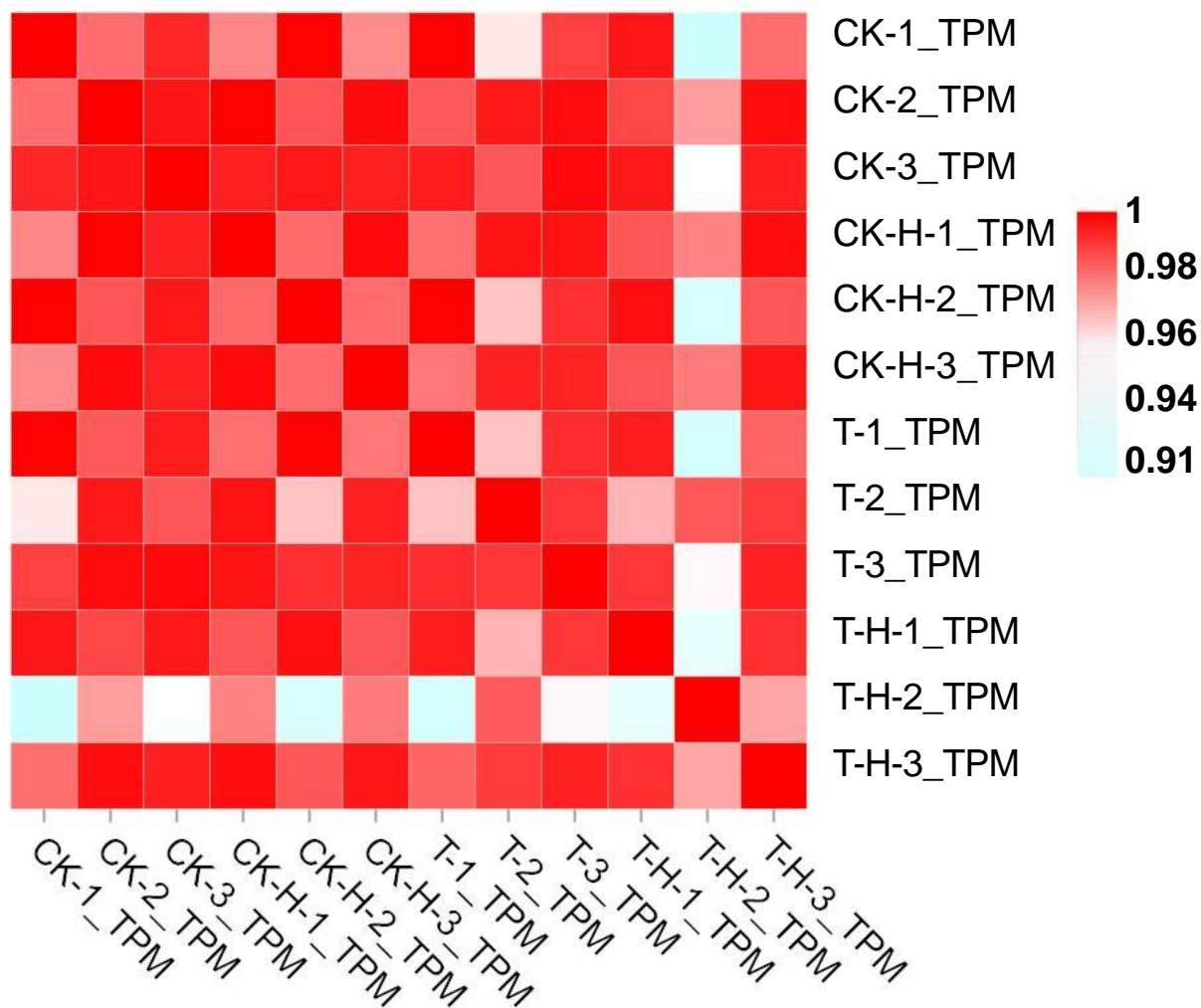

Supplement: Supplementary file 1 [file ijms-24-11085-s001.zip › Figure S1.pdf]

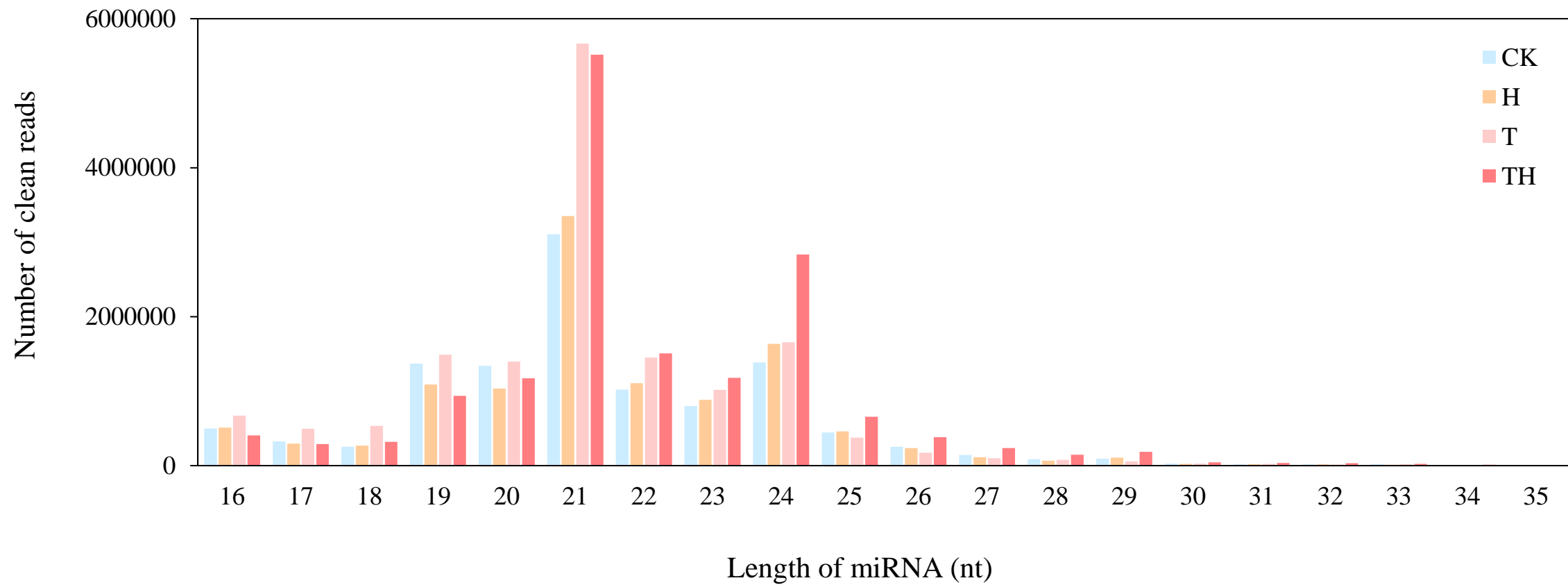

Supplement: Supplementary file 1 [file ijms-24-11085-s001.zip › Figure S2.pdf]

(A)

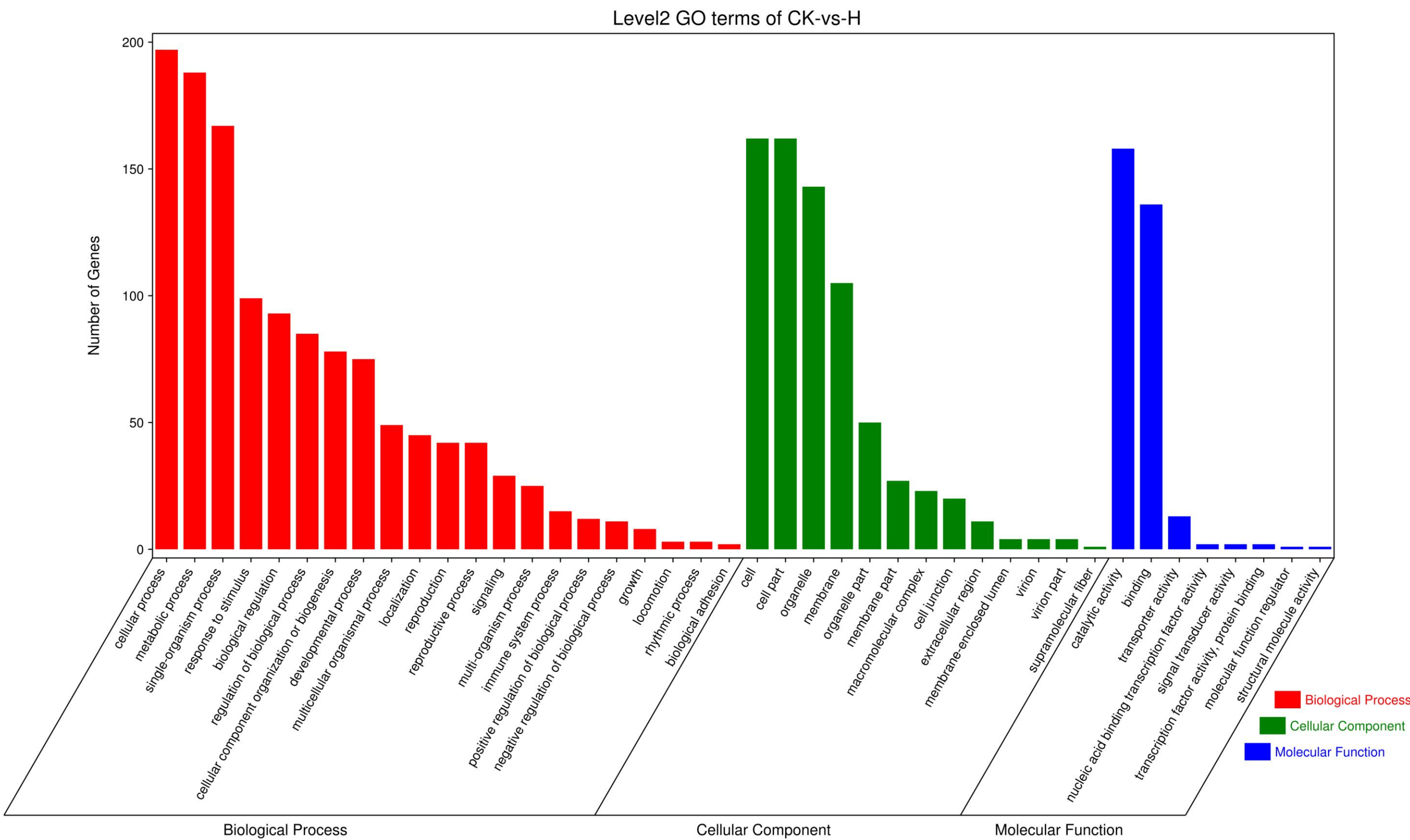

(B)

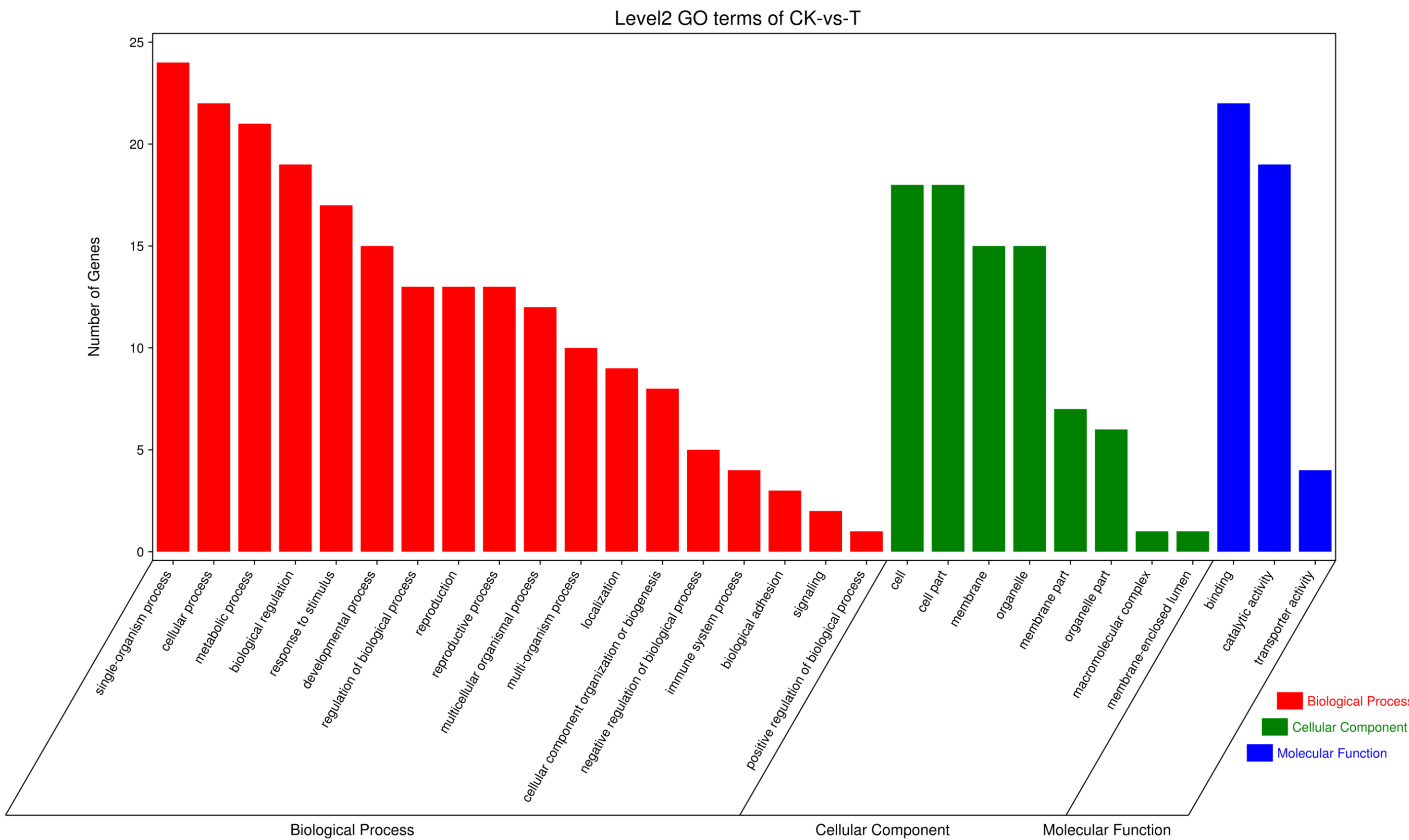

(C)

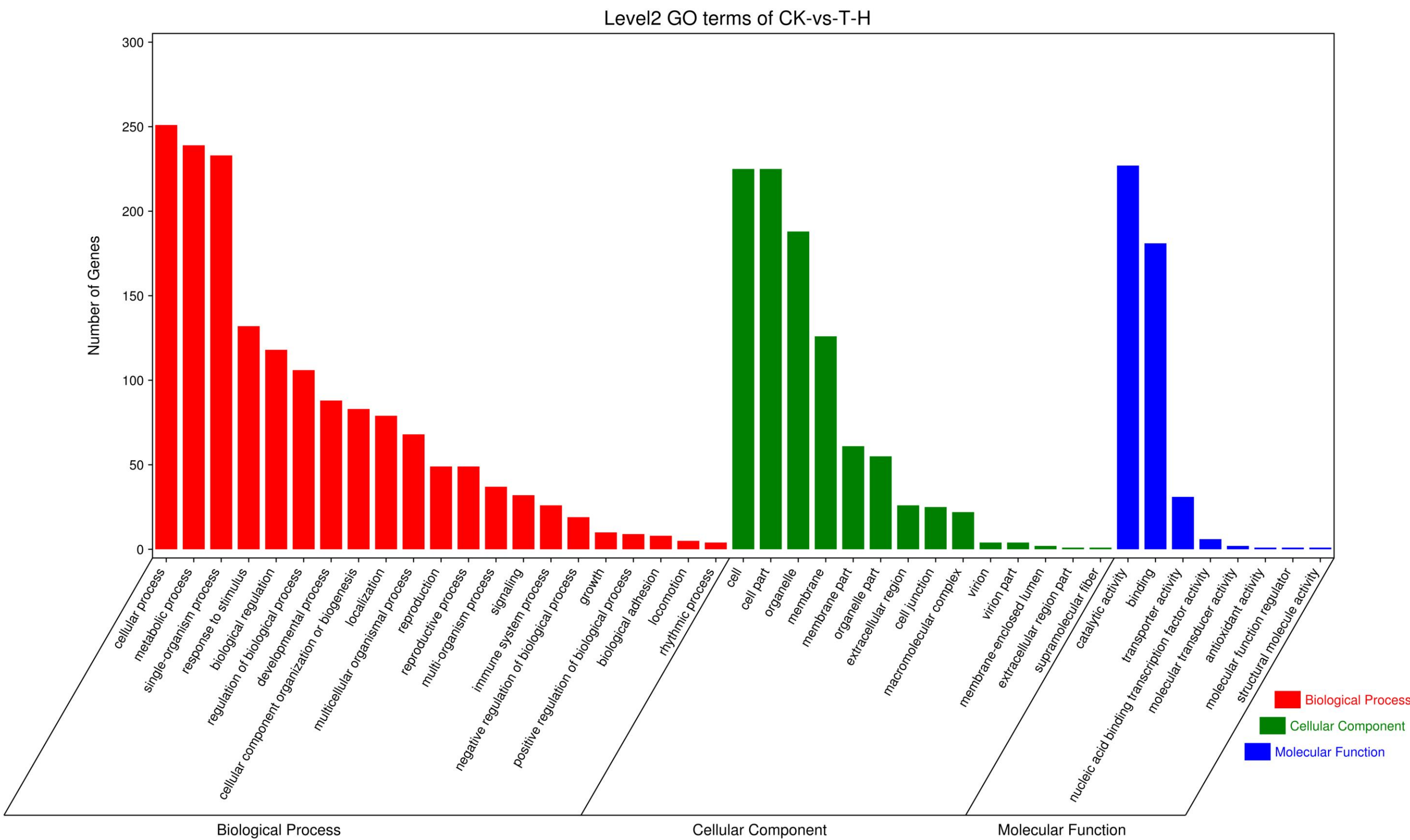

Supplement: Supplementary file 1 [file ijms-24-11085-s001.zip › Figure S4.pdf]

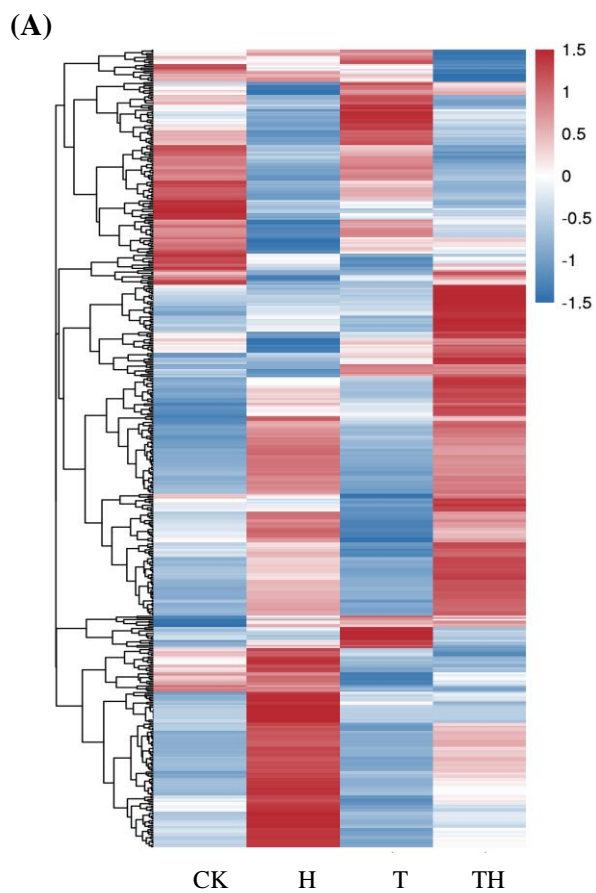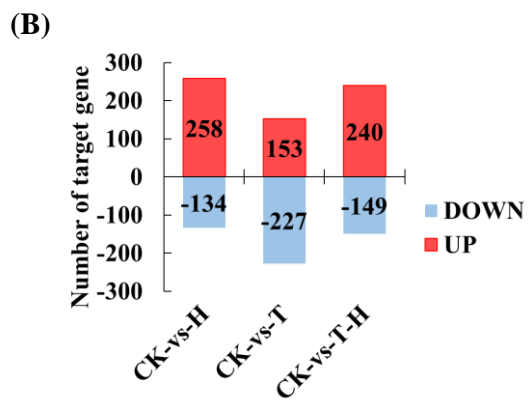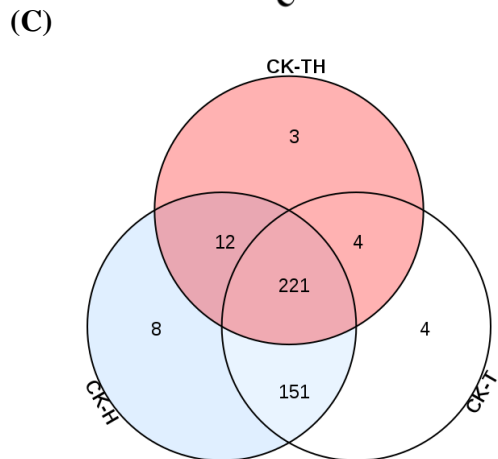

Supplement: Supplementary file 1 [file ijms-24-11085-s001.zip › Figure S5.pdf]
